# Evaluation of selective embolization of thyroid arteries (SETA) as a preresective treatment in selected cases of toxic goitre

**DOI:** 10.1186/1756-6614-2-7

**Published:** 2009-07-31

**Authors:** Marek Dedecjus, Józef Tazbir, Zbigniew Kaurzel, Grzegorz Stróżyk, Arkadiusz Zygmunt, Andrzej Lewiński, Jan Brzeziński

**Affiliations:** 1 Department of General, Oncological and Endocrine Surgery, Medical University of Lodz, Polish Mother's Memorial Hospital – Research Institute, Lodz, Poland; 2 Department of Endocrinological, General and Vascular Surgery, Copernicus Memorial Hospital, Lodz, Poland; 3 Department of Radiology, Copernicus Memorial Hospital, Lodz, Poland; 4 Department of Endocrinology and Metabolic Diseases, Medical University of Lodz, Polish Mother's Memorial Hospital – Research Institute, Lodz, Poland

## Abstract

**Background:**

in recent years, an increasing interest in the application of selective embolization of thyroid arteries (SETA) in the treatment of thyroid diseases is observed. In the present report, we analyse the value, safety and possible indications for preresective SETA in cases of large toxic goitres.

**Patients and method:**

the study group comprised 10 patients with large toxic goitre (thyroid volume 254 ± 50 mL), including one patient with cervicomediastinal goitre and one patient with anti-thyroid drug intolerance in state of overt thyrotoxicosis. All the patients underwent SETA of the superior and/or inferior thyroid arteries, followed by thyroidectomy, performed up to thirty-six hours after SETA (23.1 ± 11 h). After embolization, selective angiographies of thyroid arteries were performed to ensure that the targeted arteries had been completely occluded.

**Results and conclusion:**

in all the patients, SETA decreased blood flow through the thyroid. Preresective SETA reduced blood loss during and after thyroidectomy and decreased the operating time, but the differences were too small to justify routine applications of preresective SETA as an adjunct to surgical treatment of toxic goitre. On the other hand, SETA is a safe and minimally-invasive technique, which may become an attractive option for quick preparation to surgery in selected patients with toxic goitre, who present anti-thyroid drug intolerance or refuse radioactive iodine treatment.

## Introduction

Embolization of vascular tumours of the head, the neck and the central nervous system (CNS) has become an important adjunct to treatment of these pathologies. The technique of selective embolization has extensively been used to occlude vessels, either as an alternative to traditional therapy or when traditional therapy fails. Recently, embolization techniques have been employed more commonly and with higher precision and facility. As a result, this alternative technique has quickly evolved into the first-line therapy for many complex clinical conditions, surgical repairs of which are considered to bear high risk of various degrees. The introduction of this procedure has improved morbidity and mortality rates of patients with tumours of the head, the neck and the central nervous system, while also facilitating their removal [1,2]. The technological advances in microcatheters and new, safer embolic agents have permitted a wider range of applications of this form of treatment. In spite of this, the number of publications on the application of selective embolization of thyroid arteries (SETA) in the treatment of thyroid disorders is rather limited. The published reports are limited to the applications of SETA in treatment of thyrotoxicosis – particularly in Graves' disease [3-5] – and thyroid cancer [6-8].

On the other hand, there are some reports on the effective use of SETA in the treatment of vascular lesions of thyroid arteries [9-12]. Also numerous studies have been performed on embolization of metastases from thyroid carcinomas [13-16].

In the present report, we analyse the value of preresective SETA, applied as an adjunct to surgical treatment of selected cases of large toxic goitres. The aim of our study was to define potential indications for the application of preoperative SETA in the treatment of toxic goitre.

## Patients and methods

Ten patients with toxic goitre of second degree (WHO classification) were enrolled in the study from September 2003 to June 2006. In those patients, SETA was performed as preresective therapy. The study protocol had been approved by the Ethics Committee of the Medical University of Lodz. The patients were informed of the procedure and of all the risks and potential side effects, associated with it. Afterwards, they signed a consent form as their agreement to undergo arterial embolization.

From the Department of Surgery files a group of 20 patients with toxic goitre were selected. The group was age-, gender- and thyroid volume-matched with the group of the patients that was thyroidectomised after SETA. Groups were compared to assess whether there was any difference in the operating time and blood loss between the patients, thyroidectomised after preresective embolization and operated on without preresective embolization.

### Selective Embolization of Thyroid Arteries

The patients underwent SETA of the both superior and one of the two inferior thyroid arteries. The procedure was performed by a qualified team, using the Seldinger's technique [17]. A typical procedure required less than one hour. In brief, the patient was placed in a supine position. The inguinal pulsation point of either the left or the right femoral artery was chosen as the puncture site. A small skin incision was made under local anaesthesia (1% procaine, 2–3 mL). The puncture needle, along with a cannula, was inserted into the femoral artery through the incision. Next, the needle was removed, while the cannula remained in the vessel lumen as an entry portal for a 4 or 5F-size angiographic catheter (Vertebral – Cordis, Europe NV, the Netherlands and Multipurpose, Balton, Poland). The catheter was advanced from the femoral artery *via *the abdominal aorta and, sequentially, to both the superior and one of the inferior thyroid arteries. Migration of the catheter was visualised by a digital imaging x-ray device (Angiorey DFP-50A, Toshiba, Japan). Before embolization, a contrast medium (Ultravist 300; Schering AG, Germany) was injected into the vessels, thus allowing us to see the arteries and those regions of the thyroid to which they supplied blood. Granules, consisting of polyvinyl alcohol (PVA, Cordis Neurovascular Inc., Miami, USA), ranging from 150 to 750 μm in size, were slowly injected into the vessels. An additional step to embolize selected arteries involved the use of a non-magnetic wire coil with synthetic fibres (MReye^® ^IMWCE 35-5-8, Cook, Denmark) of appropriate size, depending on the lumen of the arteries in question. After SETA, selective angiography of the thyroid arteries – as digital subtraction angiography (DSA) images – was performed to estimate the blood flow through the gland and ensure complete occlusion of the targeted arteries. Additionally, US-scans in all the patients and CT-scans in selected patients were performed after the embolization and before surgery, allowing to analyse influence of SETA on thyroid volume and blood flow through the gland.

### Concentrations of free triiodothyronine (FT_3_), free thyroxine (FT_4_), thyrotropin (TSH), thyroglobulin (Tg), parathormone (PTH) and calcium (Ca++)

Tg, FT_3_, FT_4_, TSH, calcium, and PTH concentrations were measured by the electrochemoluminescence (ECL) method, using a Modular E170 (Roche) analyzer and appropriate kits (Roche-Diagnostics).

### Statistical analyses

Unpaired T-test was used to assess whether there was any difference in the operating time and blood loss between the patients, thyreoidectomised because of toxic goitre after preresective embolization and the thyroid volume-matched patients, thyroidectomised because of toxic goitre without preresective embolization.

## Results

**Figure 1:**
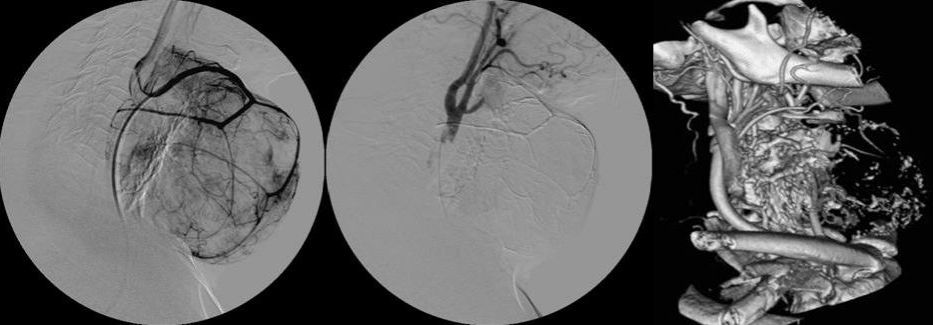
**Patient No 2: State before (on the left) and after (in the middle) embolization of the right superior thyroid artery (lateral view), and 16W angio CT-scan with 3D reconstruction 24-hours after SETA (on the right)**.

**Figure 2:**
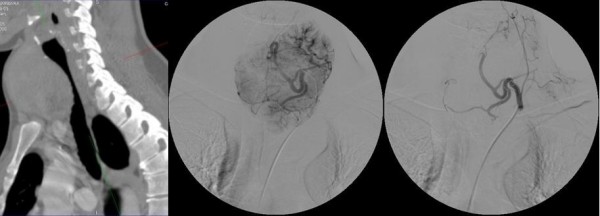
**Patient No 5: CT scan showing trachea compression (on the left) and state before (in the middle) and after SETA of the left inferior thyroid artery (on the right)**.

**Figure 3:**
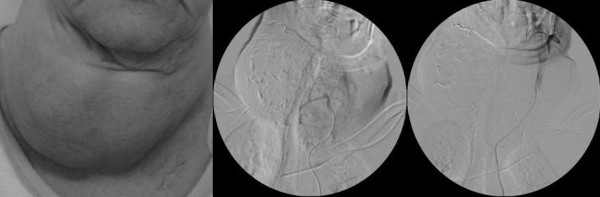
**Patient No 10: photo of the patient before the procedures (on the left), state after embolization of the right superior and right inferior thyroid arteries and during arteriography of the left inferior artery (in the middle), state after embolization of the right superior and both inferior thyroid arteries, during arteriography of the left superior thyroid artery (on the right) – the artery was not embolized because of the communication with the left sublingual artery**.

In all the patients, thyroidectomy was performed before the 36^th ^hour after SETA. Nine patients demonstrated toxic goitre at the stage of either euthyroidism or subclinical hypothyroidism (Table 1). One of the above patient had retrosternal goitre. In all the cases, SETA effectively limited blood flow through the thyroid gland – confirmed in DSA (Figures 1, 2, 3), CT scans (Figure 2) and US-scan. We retrospectively compared the operations of investigated patients with the twenty operations of toxic goitre of similar volume, performed without prior preresective SETA (patient were not randomized). The application of SETA significantly shortened the operating time and reduced drainage and blood loss during thyroidectomy (Table 2). On the other hand, preresective SETA had no influence on the rate of complications. No major complications were observed, except haematoma (2 cases), fever (2 cases) and neck pain (2 cases).

**Table 1:** Biochemical characteristics of the ten patients, who underwent preresective SETA.

**No**	**Age** *[years]*	**Thyroid volume** *[mL]*	TSH *[mU/L]*	FT_3_ *[pmol/L]*	FT_4_ *[pmol/L]*	**Time between** SETA and Tx* [hours]
1	65	240	0.45	4.33	12.90	28

2	47	230	1.27	3.76	11.15	32

3	70	215	2.16	4.57	10.30	24

4	67	210	3.68	3.78	14.46	26

5	71	220	2.48	3.25	10.89	28

6	58	250	4.16	3.12	10.42	36

7	49	248	1.86	4.10	15.16	6

8	57	285	2.46	4.27	11.86	26

9	70	260	0.98	3.12	10.12	24

10	75	380	> 0.001	18.01	237.27	1

Normal values: TSH – 0.27–4.2 mU/L; FT_3 _– 3.95 – 6.8 pmol/L; FT_4 _– 12–22 pmol/L.

*Tx-thyroidectomy

**Table 2:** Comparison of selected parameters of thyroidectomies performed with and without preresective SETA.

	**Thyroidectomy with preresective** **SETA **(N = 9)	**Thyroidectomy without preresective SETA** (N = 20)	**p**
**Operating time ***[min]*	98 ± 35	135 ± 25	< 0.01

**Blood loss ***[g]*	60 ± 12	90 ± 26	< 0.01

**Drainage in 24 hours ***[mL]*	48 ± 25	96 ± 36	< 0.01

**RLN palsy** *(constant/transitory)*	0/0	0/1	NS

**Hypoparathyroidism** *(constant/transitory)*	0/1	0/2	NS

Blood loss was calculated as difference between gauze weight before and after operation.

The patient number ten was a 75-year old female, suffering from depressive syndrome (Figure 3). She was transferred to our Department from the Endocrinology Department because of large toxic goitre, compressing the trachea. She was treated with antithyroid drugs, however, because of agranulocytosis, the treatment was stopped and, on admission to the Department of Surgery, she was in the course of ineffective treatment with lithium carbonate. In spite of evident indications to surgery, the patient was overly thyrotoxic (TSH <0.001 mIU/mL, FT_4 _237.27 pmol/L, FT_3 _18.01 pmol/L). Since the clinical condition of the patient was aggravating, with developing respiratory insufficiency, a decision was taken to perform preresective selective embolization of thyroid arteries, to be followed by immediate thyroidectomy. No significant bleeding was observed during that operation, after which, immediate amelioration was observed. The patient was discharged from the department of surgery on the 4^th ^day after the applied procedures.

## Discussion

Although the generally accepted therapies of toxic goitres are very effective in alleviating hyperthyroidism and/or thyroid ablation, there still remain some difficult cases that are not amenable to current therapeutic options. In 2002, Xiao et al. [3] proposed arterial embolization as a novel approach to thyroid ablative therapy. The authors performed selective arteriography, using Seldinger's technique [17], followed by embolization of thyroid arteries in 22 patients with Graves' disease. From that group, 14 patients remained euthyroid after SETA, 6 were operated on because of goitre and the other two needed a maintenance dose of antithyroid drug therapy [3]. A similar study was performed by Zhao et al. [4] on 28 patients with Graves' disease. From that group of patients, 22 became euthyroid, five improved and one demonstrated temporary improvement, followed by recurrence of the disease. Both groups of investigators did not observe any serious complications in any of those patients and, after a follow-up period, ranging from 12 to 24 months, they considered the procedure to be effective, minimally invasive and safe [3,4]. Interestingly enough, Zhao et al. [4] noted an increase of thyroid hormones together with a drop in TSH concentration on the third day after the embolization procedure. This is consistent with our observations in patients with differentiated thyroid cancer [8]. Moreover, we observed a massive increase of thyroglobulin concentrations and a moderate increase of free thyroid hormones, together with a fall of TSH concentration after 48 hours from SETA. In our opinion, it resulted from ischemic necrosis of the thyroid gland. Although, SETA reduces thyroid blood supply, the veins are not closed, and blood outflow remains unconstrained. In consequence, colloid from dying thyrocytes (comprising Tg, T_3_, T_4 _and, probably, other biochemical compounds) gets into circulation. This creates a potential risk of thyrotoxicosis aggravation, which may be particularly important in elderly people with ischemic heart disease and/or serious arrhythmia. Moreover, the review of the literature did not help us elucidate any potential consequences of increased serum Tg concentration. However, in one earlier study, its authors described embolization of DTC metastases to have caused massive Tg increase, which – probably – resulted in adult respiratory distress syndrome [18]. Considering the above observation, we suggested thyroidectomy to be performed till the 36^th ^hour after preresective SETA, as till that time, we did not observe in our study any significant increase in concentrations of the parameters in question [9].

Tartaglia et al. [5] also observed the increased thyroid hormones concentrations after embolization of cervicomediastinal hyperfunctioning goitre, 7 days after SETA. The goal of preresective embolization in their case was thyroid volume reduction. The authors succeeded, observing shrinkage of the thyroid gland by half of its initial volume [5]. A similar reduction of thyroid volume (from one third to one half of initial volume) was observed by Xiao et al. [3]. Neither we, nor Ramos et al., observed any significant reduction of thyroid volume after SETA [7,8]. However, as mentioned above, thyroidectomy was performed in the reported study up to the 36^th ^hour after SETA, and up to the sixth day after SETA in our previous study and in the study and the study described by Ramos et al. [7]. In the present group, the patient with retrosternal goitre was successfully operated through cervical incision after SETA. Comparing with retrospectively analysed patients, operated because of similar goitre, the patient was expected to require sternotomy. Although, after SETA, no important changes in thyroid volume were observed, we noted structural changes in the gland, which may have been responsible for the differences in its consistency, facilitating tumour removal. On the other hand, neither major nor even minor bleeding allowed performing radical manoeuvres, which could have accelerated the operation, and may have resulted in resection of the gland through cervical incision only.

The goal of SETA application in the present study was to decrease blood flow through a significant volume of thyroid tissue. Thus, decreased vascularization of thyroid tissue facilitated the removal of tumour, reducing blood loss and shortening the operation time. From the technical point of view, all the procedures were successful, effectively suppressing blood flow through the gland and resulting in a much smaller blood loss during thyroidectomy, decreased drainage and shortened operation time, although there was no difference in the rate of complications. On the other hand, the above mentioned differences were too small to justify routine applications of SETA as a pre-treatment method, even in cases of large toxic goitres. Preoperative SETA may potentially limit the risk of damaging the surrounding tissues, including the oesophagus, the parathyroid gland and recurrent laryngeal nerve. However, Tarttiglia et al. [5] reported, 30 days after SETA, a tight lesion and fibrous reaction and infiltration of giant multinucleate cells, probably caused by postembolization thyroiditis, that may have resulted in difficult recurrent laryngeal nerve preparation. Similar histological observations were described in the series by Xiao et al. [3].

The course of the treatment, applied to patient number ten, indicates potential values of SETA in quick preparation to surgery of patients with large toxic goitre and intolerance to anti-thyroid drugs (ATD) treatment. The patient was operated on in state of overt thyrotoxicosis. However, it should be underlined that the applied preresective SETA was followed by immediate thyroidectomy because of worsening respiratory insufficiency. Although the results of the treatment were excellent, the application of SETA as a pre-treatment to surgery in thyrotoxic patients with large goitres may only be recommended as the "last resort' treatment. On the other hand, recently Zhao et al. [19] published a case report of the patient with thyrotoxic crisis successfully cured with SETA. Unfortunately, considering the fact that similar patients are rarely seen in the day-to-day practice, no larger series of similar patients are likely to be investigated.

## Conclusion

Preresective SETA is a safe and minimally-invasive technique that decreases operating time and blood flow through the thyroid, minimalizing blood loss during and after thyroidectomy. This procedure may become an attractive option for quick preparation to surgery in selected patients with toxic goitre, who present ATD intolerance or refuse radioactive iodine (^131^I) treatment. Although, SETA may be an useful adjunct to surgery in selected cases of toxic goitres, it cannot be recommended as a routine treatment.

## Competing interests

The authors declare that they have no competing interests.

## Authors' contributions

MD – participated in the design of the study, drafted the project and the manuscript. JT – participated in the design of the study, was involved in SETA procedures and follow up. ZK – performed SETA procedures, participated in discussion of results and preparation of manuscript. GS – was involved in part of surgical interventions and SETA procedures. AZ – participated in patients' qualification, follow up and performed statistical analysis AL – was involved in patients' qualification, participated in the design and coordination of the study. JB – performed most of surgical interventions, participated in the design and coordination of the study. All authors read and approved the final manuscript.
